# VRK1 and AURKB form a complex that cross inhibit their kinase activity and the phosphorylation of histone H3 in the progression of mitosis

**DOI:** 10.1007/s00018-018-2746-7

**Published:** 2018-01-16

**Authors:** David S. Moura, Ignacio Campillo-Marcos, Marta Vázquez-Cedeira, Pedro A. Lazo

**Affiliations:** 1 Experimental Therapeutics and Translational Oncology Program, Instituto de Biología Molecular y Celular del Cáncer-Centro de Investigación del Cáncer, CSIC-Universidad de Salamanca, Campus Miguel de Unamuno, 37007 Salamanca, Spain; 2 Instituto de Investigación Biomédica de Salamanca-IBSAL, Hospital Universitario de Salamanca, 37007 Salamanca, Spain

**Keywords:** VRK1, Aurora kinase B, Survivin, Histone H3, Kinase, Phosphorylation

## Abstract

Regulation of cell division requires the integration of signals implicated in chromatin reorganization and coordination of its sequential changes in mitosis. Vaccinia-related kinase 1 (VRK1) and Aurora B (AURKB) are two nuclear kinases involved in different steps of cell division. We have studied whether there is any functional connection between these two nuclear kinases, which phosphorylate histone H3 in Thr3 and Ser10, respectively. VRK1 and AURKB are able to form a stable protein complex, which represents only a minor subpopulation of each kinase within the cell and is detected following nocodazole release. Each kinase is able to inhibit the kinase activity of the other kinase, as well as inhibit their specific phosphorylation of histone H3. In locations where the two kinases interact, there is a different pattern of histone modifications, indicating that there is a local difference in chromatin during mitosis because of the local complexes formed by these kinases and their asymmetric intracellular distribution. Depletion of VRK1 downregulates the gene expression of *BIRC5* (survivin) that recognizes H3-T3ph, both are dependent on the activity of VRK1, and is recovered with kinase active murine VRK1, but not with a kinase-dead protein. The H3–Thr3ph–survivin complex is required for AURB recruitment, and their loss prevents the localization of ACA and AURKB in centromeres. The cross inhibition of the kinases at the end of mitosis might facilitate the formation of daughter cells. A sequential role for VRK1, AURKB, and haspin in the progression of mitosis is proposed.

**Electronic supplementary material:**

The online version of this article (10.1007/s00018-018-2746-7) contains supplementary material, which is available to authorized users.

## Introduction

Dynamic chromosomal remodelling is an essential step in the progression of cell division. This process requires the contribution and coordination of proteins that regulate histone modifications, which are necessary for the sequential changes occurring in chromatin during mitosis, since initiation of chromosome condensation to their segregation into daughter cells. Therefore, it is likely that histones and chromatin kinases can participate in this process. Cell proliferation is a highly complex process that involves multiple sequential and coordinated steps in which the contribution of several kinases at different stages of the process has been reported. Some like cyclin-dependent kinases [1], polo-like kinases [2], and Aurora kinases [3] control cell cycle progression. Kinases that regulate chromatin condensation and additional proliferation-associated processes can also participate to complete cell division. Among these kinases is VRK1, which is activated early by mitogenic signals [4] and is regulated in cell cycle progression [5], both at the transcriptional [4] and at the kinase activity levels [4, 6], where it plays several roles [5, 7]. Among the processes regulated by VRK1 [8] are chromatin compaction in G2/M that is mediated by histone H3 phosphorylation [9], BAF1 phosphorylation that regulates nuclear envelope dynamics [10, 11], and Golgi fragmentation in mitosis [12]. After chromosome condensation in mitosis, VRK1 is mostly released from chromatin [9]. Condensed chromatin is necessary to prepare chromosomes for their realignment and correct distribution into daughter cells.

In these processes, histone phosphorylation plays a fundamental and basic role. There are at least three different kinases targeting histone H3, AURKB [13], VRK1 [6, 9], and haspin [14], but it is not known whether there is any temporal or spatial coordination between them, nor the timing of their roles. VRK1 and AURKB phosphorylate histone H3, but each preferentially targets a different residue. VRK1 phosphorylates histone H3 in Thr3 (H3–T3ph) [6, 9], and AURKB phosphorylates Ser10 (H3–S10ph) [13]. The phosphorylation of these two residues in H3 occurs in interphase, and in early mitotic cells when they are required for chromosome compaction [9], but their sequential pattern is different [15]. In mitosis, histone H3 phosphorylation in Thr3 occurs early in prophase near the nuclear envelope, moving to the pericentromeric region and disappearing late in anaphase [15]. Haspin plays a more restricted role in mitosis, and is required for metaphase chromosome cohesion [16] and alignment [17] affecting centromeres and the kinetochore [18, 19].

These kinases, VRK1 and AURKB, have different patterns of expression in proliferating cells, and their activities and functions need to be spatially and temporally coordinated. VRK1 is present in all phases of cell cycle in proliferating cells [4]; AURKB expression is restricted to mitosis [3] and is activated by autophosphorylation and interaction with other proteins such as INCEP [20]. AURKB is located in a multi-protein chromosomal passenger complex that includes survivin [21], a protein that recognizes H3 already phosphorylated in Thr3 and that is required for AURKB recruitment and localization [22]. In proliferating cells, VRK1 activates the expression of *BIRC5*/survivin [23], a subunit of the CPC complex [24]. Survivin is needed for the recruitment of AUKB to locations with H3-T3ph [25]. In this context, it is highly likely that the subcellular localization of each kinase plays an important role at different times and locations.

VRK1 is mostly a chromatin kinase and regulates histones [9, 26], but is also present in other complexes of nucleic acids, including RNA, and nucleoproteins, as exemplified by the complex with coilin [27, 28], the scaffold protein of Cajal bodies [29]. It is possible that VRK1 plays complementary roles, as a key regulator of chromatin dynamics in different situations ranging from relaxation [26] to compaction [9] at the entry of mitosis. In turn, AURKB is more restricted to chromosome dynamics during mitosis, since chromatin organization affects chromosome structure. Therefore, it is likely that a connection and complementary role between the two kinases is necessary.

In this work, we have studied the interrelationship between the activities and roles of VRK1 and AURKB in cell cycle progression.

## Results

### VRK1 can form a protein complex with AURKB

**Fig. 1:**
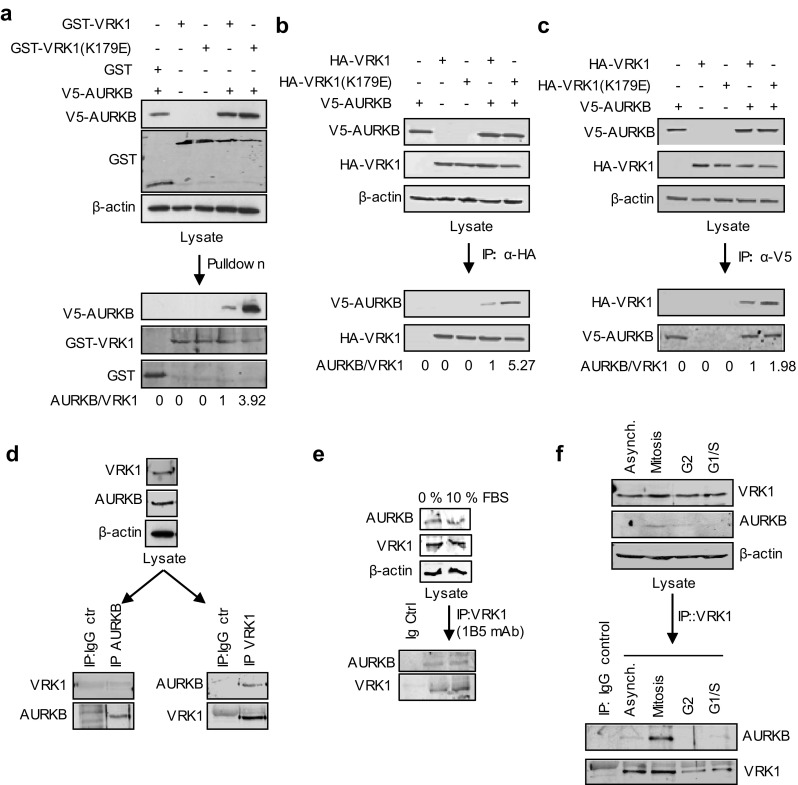
Interaction between VRK1 and AURKB. **a** Pulldown with GST–VRK1, kinase active and inactive, of V5–AURKB in HEK293T cells transfected with the indicated plasmids. **b** Immunoprecipitation of HA–VRK1 and detection of AURKB using extracts from HEK293T cells transfected with the indicated plasmids. **c** Immunoprecipitation of V5–AURKB. HEK293T cells were transfected with GST–VRK1 or HA–VRK1, WT, and kinase dead (KD), and V5–AURKB. Quantification and relative values are shown at the bottom. **d** In vitro interaction between endogenous VRK1 and AURKB. Asynchronous HeLa cells were used to immunoprecipitation of endogenous AURKB kinase (left) or endogenous VRK1 kinase (right) to detect interaction with VRK1 or AURKB, respectively. **e** Immunoprecipitation of endogenous VRK1 with 1B5 mAb in cells deprived of serum. In the immunoprecipitate, the presence of endogenous AURKB was detected in immunoblot with an anti-AURKB antibody. **f** Analysis of interaction between VRK1 and AURKB in cell cycle. Mitotic and G2 cells were obtained with thymidine/nocodazole treatment and G1/S cells with double-thymidine block. *Asynch* asynchronous cells. A detailed FACS profile of the synchronization is shown in Supplementary Fig. S1

The expression of VRK1 [4, 5] and AURKB [3] is variable in levels and activity depending on the phase of the cell cycle. Therefore, we initially determined the formation of a potential complex between these two kinases by performing pull-down and immunoprecipitation assays with overexpressed tagged proteins. In a VRK1 pull-down (Fig. 1a) and reciprocal immunoprecipitations (Fig. 1b, c), AURKB was always brought down by VRK1, and the interaction was stronger with kinase-dead VRK1 (K179E). In the immunoprecipitation of tagged AURKB, its interaction was also stronger with kinase-dead VRK1 (Fig. 1b, c). The interaction with kinase-dead VRK1 was approximately threefold stronger than with active protein. Next, we determined if the VRK1–AURKB interaction was also detectable with endogenous proteins using extracts from HeLa cells. This interaction was also detected when VRK1, a very abundant nuclear protein [30], was immunoprecipitated, but not when the minority protein, AURKB was immunoprecipitated (Fig. 1d). This observation was confirmed in asynchronous cells or serum-deprived cells in which there is a subpopulation of these two kinases that are able to form a stable protein complex, although it was only weakly detectable (Fig. 1e). It is likely that the interaction might be dependent on cell cycle and occur only in a cell subpopulation. Therefore, cells were studied in different phases of the cell cycle. The interaction between VRK1 and AURKB was mainly detected in mitotic cells (Fig. 1f), at the time when AURKB expression reaches its maximum level.

### VRK1 and AURKB localization and interaction in cell cycle progression

**Fig. 2:**
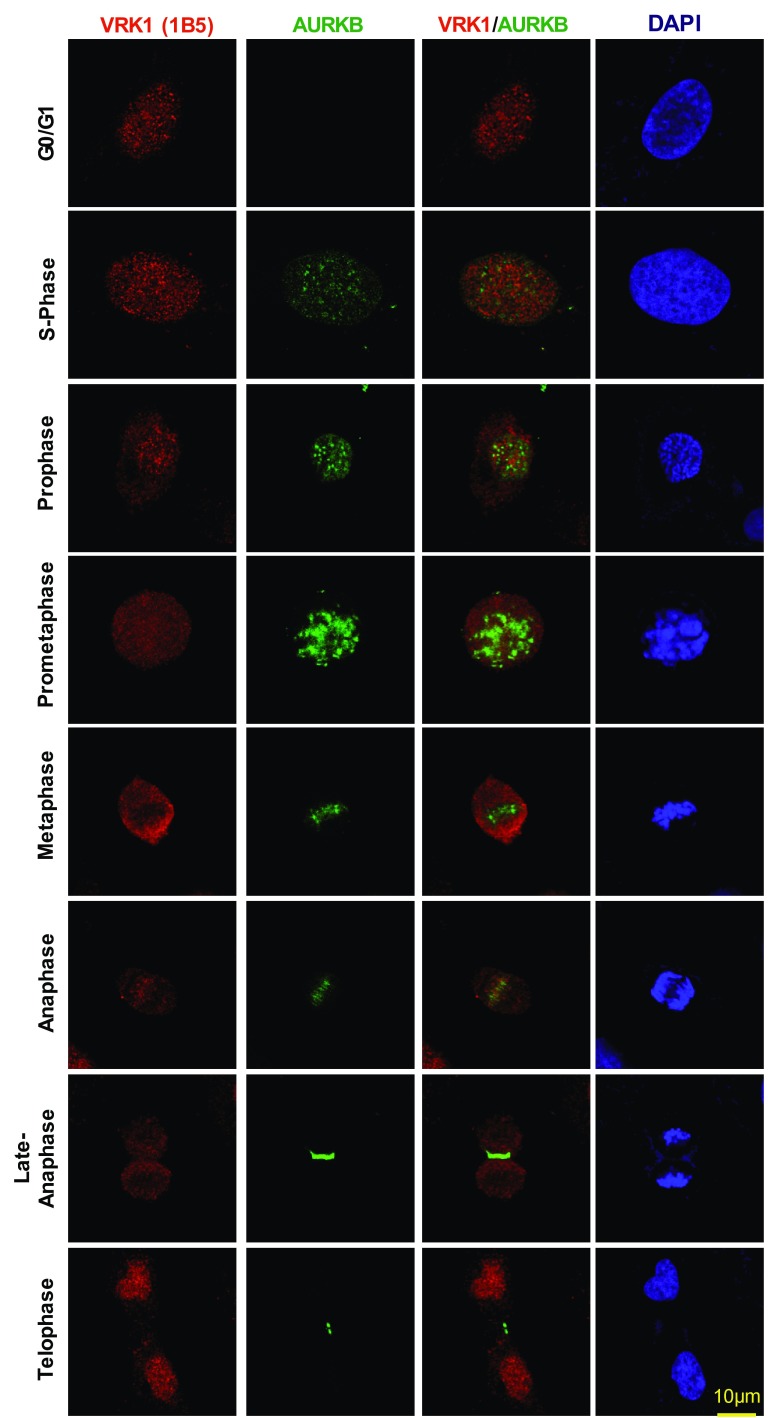
Subcellular localization of VRK1 and AURKB in mitosis. VRK1 and AURKB localizations during cell cycle progression and mitosis. 24 h after plate the cells, U2OS cells were treated with serum-free medium for 72 h, to arrest the cells at G0/G1, or with double-thymidine block to arrest cell cycle at S-phase, or with double-thymidine followed nocodazole treatment to arrest cells at G2/early mitosis, or after double-thymidine and nocodazole treatment, released from the arrest during 360 min. The known AURKB distribution in mitosis is also used as an internal control. In immunofluorescence, AURKB was detected with rabbit monoclonal anti-AURKB (N-term) antibody. Human VRK1 was detected using mouse monoclonal anti-VRK1 antibody. The flow cytometry profile of synchronized cells and their release is shown in Fig. S1. A more detailed image with additional time points in the thymidine/nocodazole release is shown in Supplementary Fig. S2. Immunofluorescence experiments were performed three times

VRK1 is a regulator of multiple steps, early and late, in cell division [5]. To determine how VRK1 and AURKB proteins are distributed along cell cycle progression, cells were arrested with thymidine–nocodazole followed by their release to identify the sequential steps of mitosis and determine the localization of both proteins, which was determined by confocal immunofluorescence. Therefore, VRK1 is always present in cells in all phases of cell cycle progression, including mitosis when there is a disassembly of the nuclear envelope. VRK1 colocalizes with chromatin in interphase, but not from prophase to telophase (Fig. 2), consistent with its early contribution to facilitate chromatin condensation [9], and its signal did not overlap with AURKB (Fig. 2). AURKB is also a control for its known localization in mitosis. Once chromosomes are condensed, VRK1 is no longer on chromatin in metaphase, anaphase, and early telophase (Fig. 2). Therefore, after chromatin condensation, and from prophase, there is no detectable overlap of VRK1 with condensed DNA. In mitosis, AURKB is expressed during prometaphase in arrested cells, and following nocodazole release, it switches from binding to chromatin in centromeres to remaining in the central spindle as chromosomes progress through anaphase and is required for mitotic exit. Only a minor colocalization of VRK1 and AURKB is detectable in anaphase in the central spindle. VRK1 is later relocated to chromatin in telophase (Fig. 2, Supplementary Fig. S2). These data indicated that the formation of a VRK1/AURKB protein complex constitutes a minor subpopulation of both proteins at some specific locations on chromatin, and which might have relevance for the temporal coordination of events at these restricted localizations during mitotic progression.

### VRK1 and AURKB cross inhibit their kinase activity and the specific phosphorylation of histone H3 and p53

**Fig. 3:**
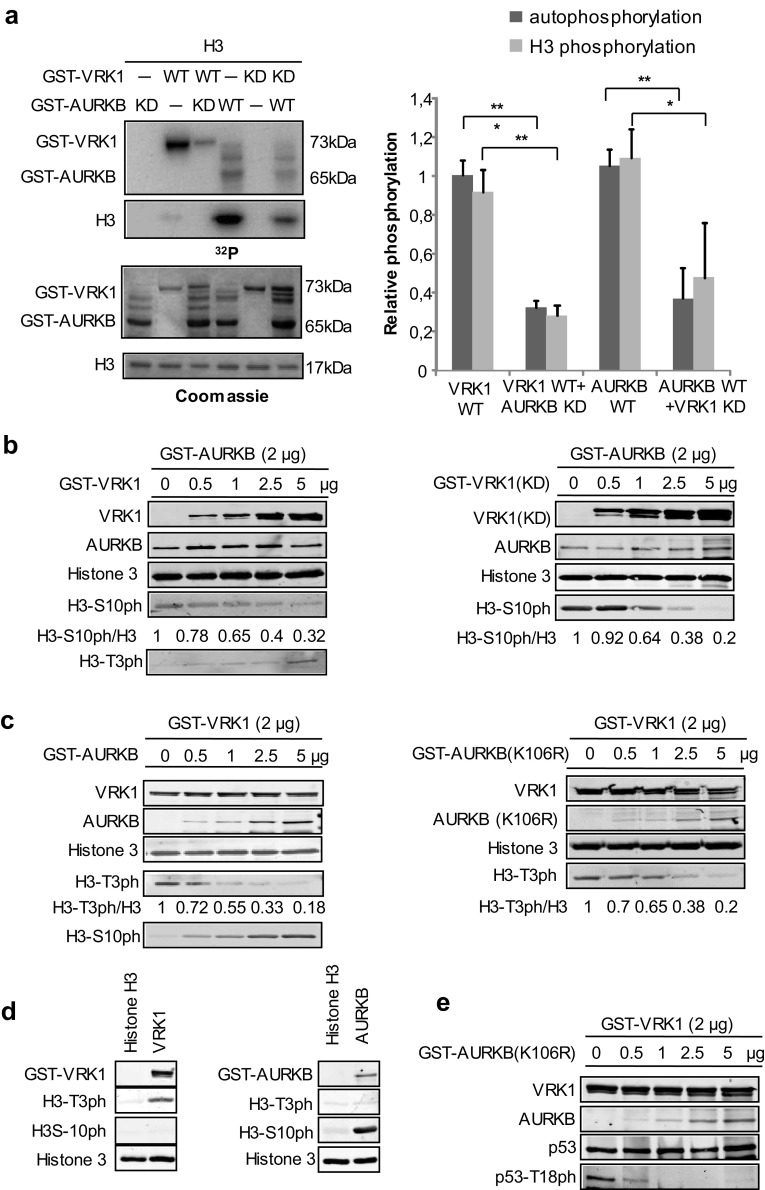
VRK1 and AURKB cross inhibit their kinase activity. **a** In vitro kinase assay with GST–VRK1 and GST–AURKB, and their kinase-dead mutants, and human histone H3 as a substrate. Proteins were incubated for 30 min at 30 °C in the presence of 5 µM ATP and 5 µCi ^32^P[ATP]. The quantification of the level of phosphorylation is shown in the graph to the right. Student’s test, *P < 0.05, **P < 0.005, ***P < 0.0005. **b** Increasing amounts of VRK1 (left panel) or kinase-dead (K179E) VRK1 (right panel) inhibit the phosphorylation of Histone 3 phosphorylation in Ser10 by AURKB. There is an inverse correlation between H3–S10ph and H3–T3ph as the ratio of AURKB/VRK1 changes. **c** Increasing amounts of ARKB (left panel) or kinase-dead ARKB (K106R) (right panel) inhibit the phosphorylation of Histone 3 phosphorylation in Thr3 by VRK1. There is an inverse correlation between H3–T3ph and H3–S10ph as the ratio of VRK1/AURKB changes. **d** (left) VRK1 directly phosphorylates Thr3 in histone H3. **d** (right) AURK directly phosphorylates Ser10 in histone H3. In vitro kinase assay with cold ATP. GST–VRK1 (pGEX–GST–VRK1) or GST–AURKB (pGEX–GST–AURKB) was incubated with human histone 3 at 30 °C for 30 min. H3–T3p was detected using a rabbit polyclonal anti-phospho-H3T3 antibody. H3–S10p was detected using a rabbit polyclonal anti-phospho-H3S10. Total histone 3 was detected using a rabbit polyclonal anti-histone 3 antibody, and GST-fusion proteins were detected using a mouse monoclonal anti-GST antibody. **e** Increasing amounts of kinase-dead AURKB (K106R) inhibit the phosphorylation of p53 in Thr18 by VRK1. Immunofluorescence experiments were performed three times

The formation of a complex between VRK1 and AURKB indicates that it is possible that their kinase activities or specificities of phosphorylation will be affected. Therefore, it was first determined in an in vitro radioactive kinase assays if phosphorylation of histone H3 could be inhibited. For this aim, different combinations of wild-type and kinase-dead (KD) forms of either VRK1 or AURKB were used. Autophosphorylation of VRK1 was inhibited by kinase-dead AURKB (Fig. 3a), and phosphorylation of histone H3 by AURKB was inhibited by kinase-dead VRK1 (K179E) (Fig. 3a). The phosphorylation of AURKB by VRK1 and VRK1 by AURKB was tested using a kinase-dead form as substrate. Neither of these kinases directly phosphorylated the other in vitro kinase assays (Supplementary Fig. S3).

VRK1 phosphorylates histone H3 mainly in Thr3 [26], and AURKB phosphorylates histone H3 in Ser10 [13]. Therefore, a kinase assay was performed in which the specific phosphorylation of each histone H3 residue was determined, using either an active kinase in the presence of the other kinase in its active or kinase-dead form. The specific phosphorylation of histone H3 in Ser10 by AURKB was inhibited in a dose-dependent manner by both VRK1 (Fig. 3b, left) and its kinase-dead form (Fig. 3b, right). There is an inverse correlation between H3–S10ph and H3–T3ph as the ratio of AURKB/VRK1 changed. Similarly, the phosphorylation of histone H3 in Thr3 by VRK1 was inhibited in a dose-dependent manner by increasing amounts of AURKB (Fig. 3c, left) or kinase-dead AURKB (K106R) (Fig. 3c, right). Similarly, there is an inverse correlation between H3-T3ph and H3-S10ph as the ratio of VRK1/AURKB proteins varied. The specific phosphorylation of Histone H3 in Thr3 or Ser10 by VRK1 or AURKB, respectively, was confirmed by in vitro kinase assays with purified proteins (Fig. 3d). The GST moiety does not inhibit the effect of each kinase on H3 phosphorylation (Supplementary Fig. S4).

Furthermore, the specific phosphorylation of p53 in Thr18 by VRK1 [31–33] is also inhibited in a dose-dependent manner by kinase-dead AURKB (Fig. 3e). This indicates that the inhibition of VRK1 by AURKB is more general and can affect other substrates.

These results indicated that in those subcellular locations where the two kinases interact forming a complex, they are able to cross inhibit their respective kinase activities and phosphorylations of histone H3 or p53. P53 is phosphorylated by both VRK1 [31] and AURKB [34], which have opposing roles in p53 stability [34].

### VRK1 and AURKB interaction inhibits phosphorylation of histone H3 in vivo

**Fig. 4:**
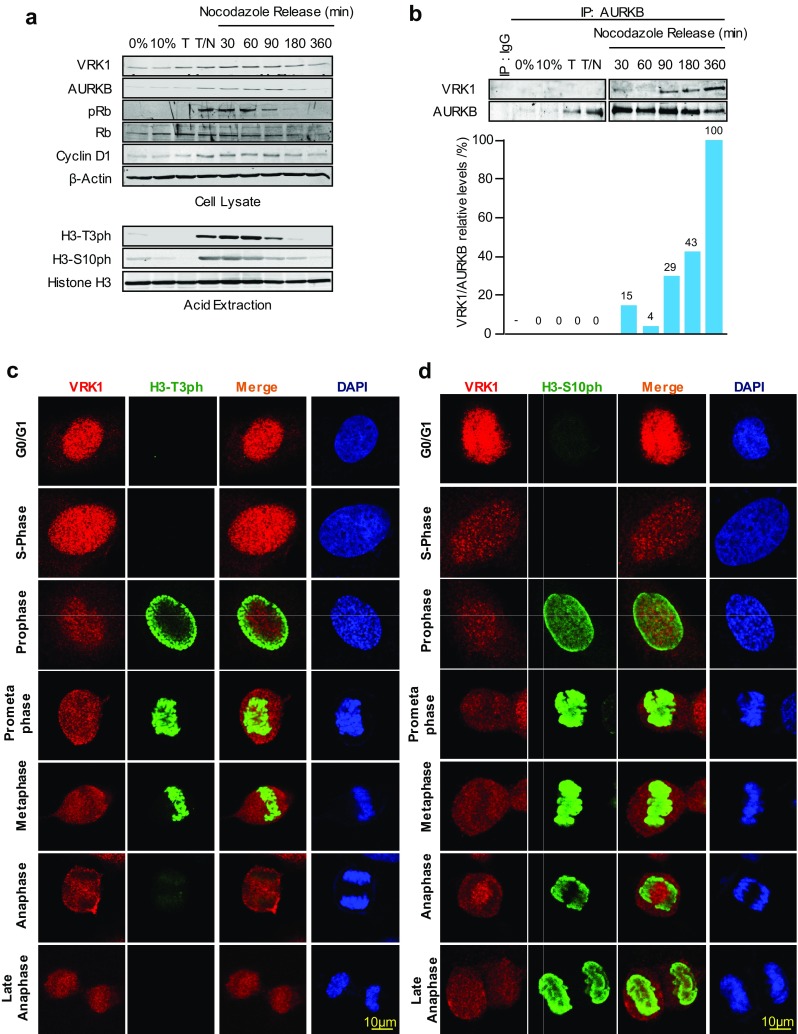
VRK1–AURK complex and histone H3 phosphorylation in cell cycle progression. **a** Expression of VRK1, AURKB, histone H3, and cell cycle markers in different phases of the cell cycle. U2OS cells were synchronized with thymidine/nocodazole treatment, which was followed at different time points after the blockade release (top panel). The detection of H3 phosphorylated in Thr3 or Ser10 (bottom panel) using acid extraction of histones in immunoblots. **b** Detection of the AURKB/VRK1 protein complex in mitosis with rabbit monoclonal anti-AURKB (N-term) antibody (Epitomics) or with a rabbit polyclonal antibody as control. At the bottom is shown the quantification of the relative amount of VRK1 bound to AURKB. **c** Detection of histone H3-T3ph in individual synchronized cells, and after nocodazole release, by confocal immunofluorescence. **d** Detection of histone H3-S10ph in individual synchronized cells, and after nocodazole release, by confocal immunofluorescence. The known pattern of H3-S10ph in mitotic progression is also an internal control. AURKB was detected using a rabbit monoclonal anti-AURKB (N-term) antibody. Human VRK1 was detected anti-VRK1 antibody (1F6, western blots; 1B5 immunofluorescence). H3T-3ph (rabbit polyclonal, Millipore), H3S10ph (rabbit monoclonal, Millipore). Histone H3 (rabbit polyclonal, Cell Signaling). The flow cytometry profile is shown is Supplementary Fig. S1. A detailed image of the different time points in the thymidine/nocodazole release is shown in Supplementary Fig. S2

VRK1 is expressed in all phases of the cell cycle, and its activity reaches a peak in G2/M that is required for chromosome condensation [4, 23]. AURK expression is restricted to mitosis, mainly in metaphase and anaphase [3]. Therefore, the phosphorylation pattern of histone H3 in Ser10 and Thr3, which are targeted by AURKB [13] and VRK1 [35], respectively, was studied in the synchronized population by immunoblot to determine whether histone 3 phosphorylation was affected as a result of the VRK1–AURKB interaction during cell cycle progression. In synchronized cells and following nocodazole release, there was a loss of histone 3 phosphorylation in Thr3 at 90 min and of Ser10 at 180 min (Fig. 4a). The phosphorylation of both H3 residues was not detected at 360 min. The loss of H3–Thr3 phosphorylation preceded that of H3–Ser10 and coincided with the time at which there is an increase in AURKB levels in mitosis, and both were not detectable at 360 min (Fig. 4a), when the interaction between AURKB and VRK1 reached its maximum as demonstrated by immunoprecipitation (Fig. 4b). The complex VRK1–AURB is mainly detected after nocodazole release (Fig. 4b), in which both H3 phosphorylations and phospho-Rb are lost (Fig. 4a), which might be a signal to facilitate the completion and exit of mitosis. The increased detection of the AURKB–VRK1 complex (Fig. 4b) by immunoprecipitation coincides with the timing for the loss of H3T3ph followed by H3S10ph loss (Fig. 4a) are detectable. This sequential loss of phosphorylation and the precedence of a reduction in Thr3 over Ser10 phosphorylation in mitosis were confirmed by confocal immunofluorescence for H3 residues Thr3 (Fig. 4c) and Ser10 (Fig. 4d). H3–T3ph is localized in the periphery in prophase and moves to condensed chromosomes in prometaphase (Fig. 4c). In prophase, histone H3-S10ph presents a more diffuse pattern that does not overlap with H3–T3ph, except for some in the periphery (Fig. 4c). In prometaphase and metaphase, both H3 phosphorylations colocalize with condensed chromosomes. However, in anaphase, phosphorylation in H3–T3ph was lost in the cell (Fig. 4c), which corresponds to the time when the interaction between VRK1–AURKB can occur (Fig. 2). At this time, H3–S10ph remained associated with chromatin (Fig. 4d). The timing of these phosphorylation patterns in the phases of cell cycle (Fig. 4c, d) is consistent with the phosphorylation patterns detected in the population by immunoblots (Fig. 4a). VRK1 was not detected on chromatin in the later phases of the cell cycle confirming the previous observation (Fig. 2), which was expected, because VRK1 is ejected from chromatin following the phosphorylation of H3 in Thr3 after initiation of chromatin condensation in mitosis [9].

### Depletion of VRK1 causes a loss of *BIRC5* (survivin) gene expression

**Fig. 5:**
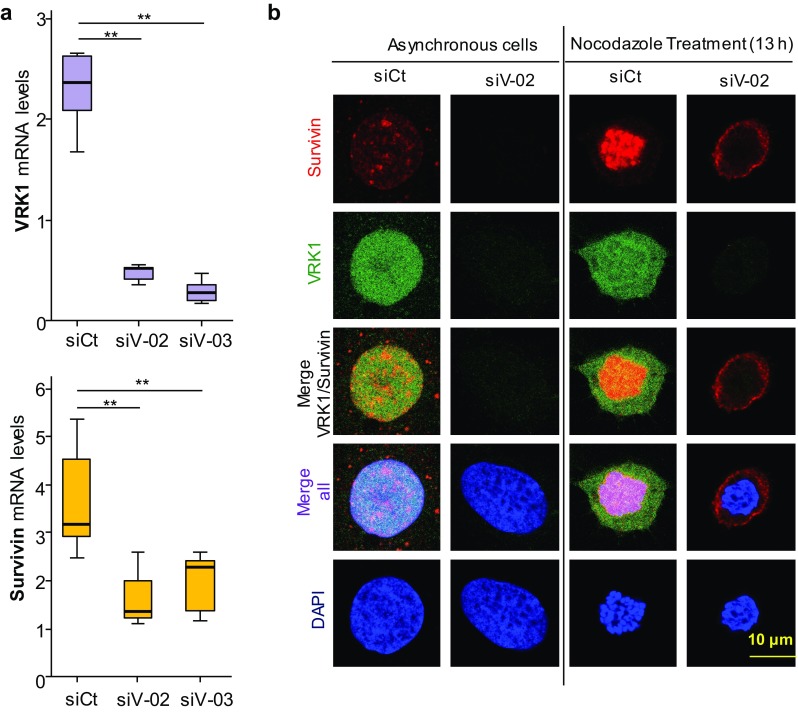
VRK1 is required for expression of endogenous *BIRC5* (survivin) gene expression. **a** VRK1 depletion (top) cause a loss of expression of survivin (bottom) in U2OS cells. Similar result was obtained in HEK293T cells (Supplementary Figure S4). The level of RNA was determined by qRT-PCR. Each experiment was independently performed three times. In each experiment, values were also determined in triplicates. **P < 0.01. *siCt* siControl, s*iV-02* siVRK1-02, *siV-03* si-VRK1-03. **b** VRK1 depletion causes a loss of expression of *BIRC5* (survivin) in U2OS cells detected by immunofluorescence. U2OS cells were either asynchronous or arrested with nocodazole. In both, depletion of VRK1 was performed with siVRK2-02

VRK1 is an early participant for the initiation of cell cycle progression, including exit from G0, passing the G1 restriction point and facilitate chromosome condensation to initiate mitosis [9]. Moreover, VRK1 is able to directly regulate the short proximal promoters of *BIRC5* (survivin) and *CDK2* genes in luciferase assays [23], two early genes. Therefore, we tested whether depletion of *VRK1* gene expression performed with two different siVRK1 oligonucleotides in two different cells lines was able to downregulate the expression of endogenous human *BIRC5* gene coding for survivin. VRK1 depletion caused a significant reduction of survivin RNA that was detected by qtRT-PCR in U2OS (Fig. 5a) and HEK293T (Supplementary Fig. S5) cells. Therefore, VRK1 regulates two components needed for the recruitment of AURKB in mitosis. Survivin is a reader of H3-Thr3ph [25] mediated by VRK1 early in mitosis. The presence of survivin in cells was determined in different situation, asynchronic and nocodazole arrested cells. Depletion of VRK1 resulted in a loss of survivin (Fig. 5b). The H3–Thr3ph protein interacts with survivin forming a complex that is required for the sequential recruitment and localization of AURKB to centromeres [25]. In the absence of VRK1, the survivin-H3-T3ph complex cannot be formed.

### Depletion of VRK1 alters the centromeric localization of AURKB in nocodazole-treated cells

**Fig. 6:**
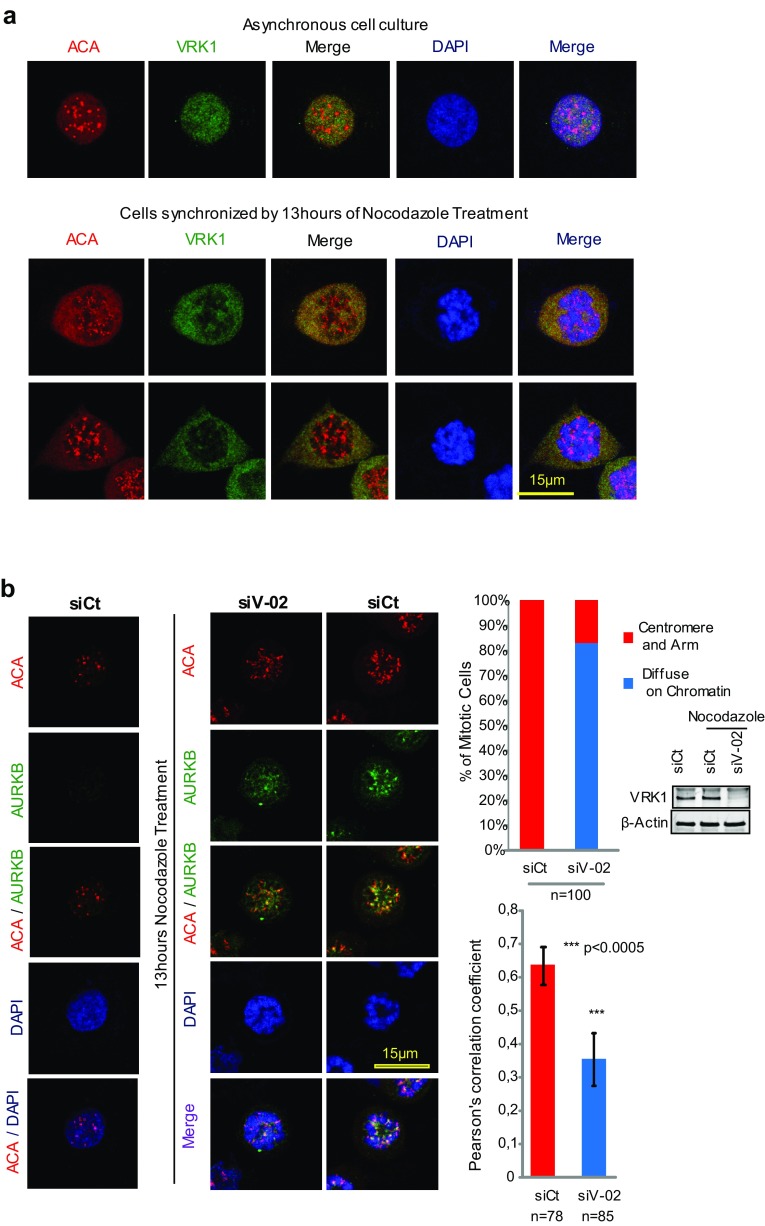
Effect of VRK1 on the centromeric localization of AURKB and ACA. **a** VRK1 in U2OS cells does not colocalize with ACA in non-synchronic cells (top panel) or in cells synchronized and arrested with nocodazole (bottom panel). **b** Colocalization of AURKB and ACA on centromeres is affected by the knock down of VRK1, indicating that it interferes with centromere recruitment. The U2OS cells were treated during 13 h with nocodazole. AURKB was detected using a rabbit monoclonal anti-AURKB antibody and ACA was detected using a human anti-ACA antibody. Immunofluorescence experiments were performed three times. The number of cells is indicated in the figure (additional individual cells are shown in Supplementary Figure S6). The quantification of the AURK and ACA signals, and the DAPI signal (DNA) were quantified with the confocal software and their overlap is shown in the graphs to the right. A total of 100 cells were counted taking into account the distribution of AURKB on centromeres and chromosome arm or diffused on the chromatin (Chi-square statistic is significant at *P* < 0.01). Colocalization studies were performed by two distinct methods: the first by analyzing the images and the distribution of AURKB on centromeres and chromosome arms, or diffused on the chromatin, and the second calculating the Pearson’s correlation coefficient value separately for each cell. This coefficient gave us the value of overlapped red and green pixel in each cell. The Pearson’s correlation coefficient value was calculated separately for each cell, giving the value of overlapping red and green pixels in each cell (Student’s test: *P < 0.05, **P < 0.005, ***P < 0.0005). To the right is shown the reduction in the level of VRK1 after its knockdown by immunoblot

Although VRK1 is not a centromere protein in mitosis, as shown by the lack of a colocalization with ACA (anti-centromere antibody) in asynchronous cells (Fig. 6a). This role as an early signal in cell division [5] might affect either the recruitment or the localization of some of the components, which are later required for the assembly of centromeres, particularly if they are VRK1-dependent. In asynchronous cells, AURKB was not detectable (Fig. 6b, left). In cells arrested by treatment with nocodazole, there is already a significant level of AURKB protein located on centromeres of chromosomes (Fig. 6b, right). The knockdown of VRK1 resulted in a delocalization of AURKB from centromeres, without any effect on its level. For this aim, we determined the colocalization of AURKB and ACA (Fig. 6b), a centromeric marker, and the effect that VRK1 depletion on their organization. In nocodazole-treated cells, ACA and AURKB colocalize, forming clearly detectable granules, which correspond to centromeres (Fig. 6b, right). Following VRK1 knockdown, the colocalization of ACA and AURKB on centromeres was significantly reduced (Fig. 6b, middle). The quantifications of the colocalizing signals are shown in the graph, in which a loss of correlation between the AURK and ACA signals was detected by Pearson analysis (Fig. 6b, right). This indirect effect of VRK1 supports the dependence of later steps in cell cycle progression on earlier VRK1 effects in cell cycle progression. These are the expected effects dependent on VRK1, because depletion of VRK1 causes a loss of survivin expression and of H3-T3ph, which are later required for the recruitment of AURKB to centromeres in mitosis.

### Phosphorylation of histone H3 in Thr3 by VRK1 is required for centromere formation

**Fig. 7:**
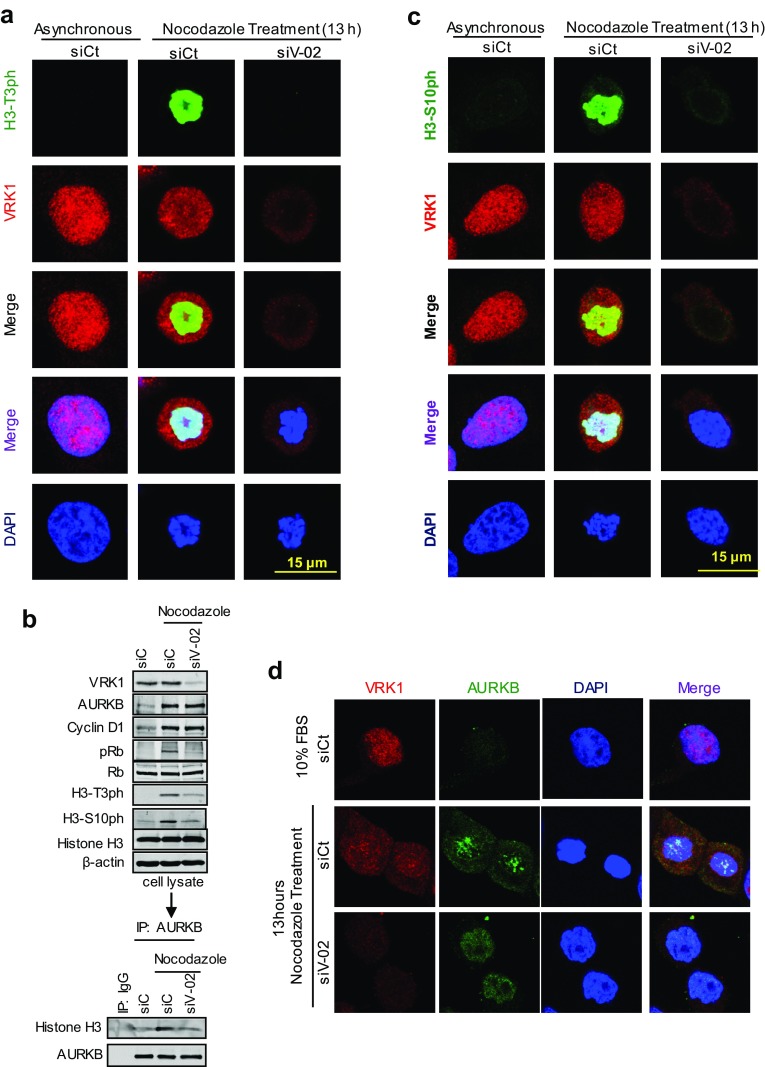
VRK1 alters the phosphorylation of H3 in Thr3 and the localization of AURKB in centrosomes. **a** VRK1 downregulation affects the phosphorylation of histone H3 on Thr3 residue. At the top are shown non-synchronized cells. At the bottom are shown U2OS cells in which siControl (siCt) or si-VRK1 (siV1-02) was performed, and 2 days later, cells were treated with nocodazole for 13 h. H3-T3ph was detected using a rabbit monoclonal anti-H3-T3ph and VRK1 was detected using a mouse monoclonal anti-VRK1 (1B5) antibody. **b** Depletion of VRK1 in nocodazole-treated cells caused a reduction in phopho-Rb and phosphorylation of H3 in Thr3 and Ser10. The relative values of H3 coprecipitating with AURKB are the mean of three experiments, and value 1 is the reference in unsynchronized cells. **c** VRK1 downregulation affects the phosphorylation of histone H3 on Ser10 residue. At the top are shown non-synchronized cells. At the bottom are shown U2OS cells in which siControl (siCt) or si-VRK1(siV1-02) was performed and 2 days later cells were treated with nocodazole for 13 h. H3-S10ph was detected using a rabbit monoclonal anti-H3S103ph and VRK1 was detected using a mouse monoclonal anti-VRK1 (1B5) antibody. **d** Depletion of VRK1 altered the localization of AURKB on centromeres in nocodazole-treated cells. The U2OS cells were treated during 13 h with nocodazole. Immunofluorescence experiments were independently performed three times

AURKB forms a complex with a histone H3 that is already phosphorylated in Thr3, and this binding of AURKB is mediated by survivin, which is regulated by VRK1 [23], as part of its recruiting mechanism [25]. Therefore, it is likely that the effect of VRK1 on the centromeric localization of AURKB might be indirect, and a consequence of the loss of Histone H3 phosphorylation in Thr3 by VRK1 at the initiation of mitosis. In this context, we first tested the effect of VRK1 knockdown by immunofluorescence. VRK1 depletion resulted in a loss of H3 phosphorylation in Thr3 (Fig. 7a), which also resulted in the loss of AURKB interaction with H3 (Fig. 7b) and consequently prevented the phosphorylation of H3 in Ser10 (Fig. 7c), a likely consequence of the loss of *BIRC5*/survivin gene expression that is also VRK1-dependent [23] (Fig. 5). The loss of both H3 phosphorylations was confirmed in immunoblots (Fig. 7b). Furthermore, depletion of VRK1 affected the localization of AURKB in centromeres. Therefore, a phosphorylation that is required for the binding of AURKB to H3 might alter its local position in chromatin. In mitosis-arrested cells, the loss of VRK1 resulted in a redistribution of AURKB, instead of having the granular aspect of centromeres in prophase (Figs. 2, 7d). Depletion of VRK1 resulted in a diffuse redistribution of AURKB on chromatin (Fig. 7d).

### The kinase activity of VRK1 is necessary to rescue H3–T3 phosphorylation, survivin expression, AURKB localization, and H3–S10 phosphorylation

**Fig. 8:**
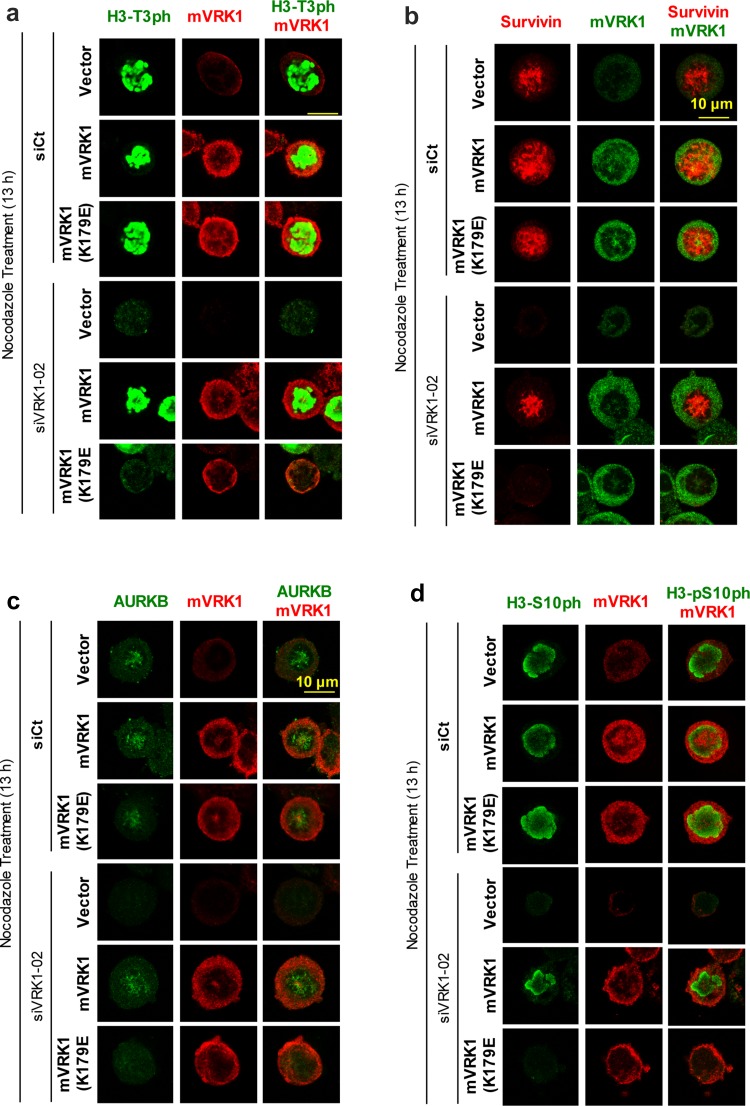
Rescue of H3 phosphorylation, survivin, and ARKB with kinase-active murine VRK1. In U2OS cells, endogenous human VRK1was knocked-down with si-VRK1-02 and transfected with murine VRK1 (mVRK1) kinase active and inactive (mVRK1-K179E). Cells were treated with nocodazole for 13 h. The phosphorylations of histone H3-T3ph (**a**) and presence and localization of survivin (**b**) and ARKB (**c**) and the presence of H3-S10ph (**d**) determined by immunofluorescence, which represents their sequential order. Cells containing murine VRK1 were identified with an antibody against the myc epitope. The complete figure is shown as Supplementary Figure S7

The phosphorylation of H3–Thr3 and expression of survivin are necessary steps for the later recruitment and localization of AURKB in mitosis. To determine if these effects are dependent on the activity of VRK1, a rescue experiment was performed. The endogenous human VRK1 was knocked-down and these cells were transfected with plasmids expressing murine VRK1 active, or kinase-dead (K179E), which are insensitive to siRNA targeting human VRK1 [28]. The cells were arrested with nocodazole. The phosphorylation of histone H3 in Thr3 and Ser10, and the expression of survivin and AURKB were determined in rescued cells. Kinase active, but not the kinase-dead mVRK1(K179E) mutant, was able to recover the phosphorylation of H3 in Thr3 (Fig. 8a), the expression of survivin (Fig. 8b) and AURKB (Fig. 8c), and the H3-Ser10 phosphorylation (Fig. 8d). Therefore, the kinase activity of VRK1 is necessary for the initial phosphorylation of H3-Thr3 and subsequent expression of survivin, both required for the later recruitment of AURKB in mitosis and the phosphorylation of histone H3 in Thr10 by AURKB.

### VRK1 contributes to AURKB stability

**Fig. 9:**
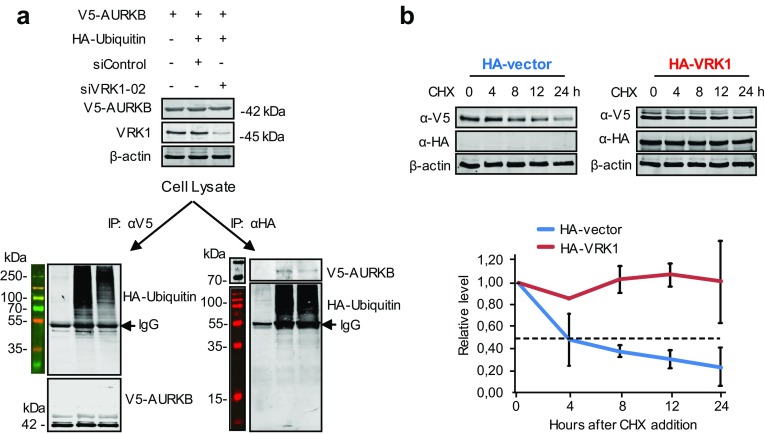
VRK1 contributes to the stability of AURKB. **a** 293T cells were transfected first with SiControl or SiVRK1-02 and 24 h later transfected using PEI with AURKB (pcDNA3.1/nV5–DEST–AURKB) in combination with V5 and with active HA-ubiquitin (pSSK–HA––Ubiquitin). The immunoprecipitated proteins were determined in immunoblots. **b** The 293T cells were transfected with plasmids expressing HA–VRK1 (pCEFL–HA–VRK1) and V5-tagged AURKB. 72 h after transfection and overexpression, the 293T cells were treated with 50 µg/mL of cycloheximide and aliquots were taken at different time points during 24 h for protein level determinations. The two experiments were performed three times and the error bars represent the standard deviations. The dashed line represents the 50% level

VRK1 contributes to the stability of several protein by protecting them from ubiquitin-mediated degradation, as is the case of p53 [31], NBS1 [36], or coilin [28]. AURKB is degraded at the end of mitosis by ubiquitin-mediated degradation in the proteasome [24, 37, 38]. Therefore, it was tested whether depletion of VRK1 might affect the stability of AURKB. For this aim, cells were transfected with plasmids expressing V5–AURKB and HA–ubiquitin. Depletion of VRK1 reduced the amount of ubiquitinated AURKB as detected by reciprocal immunoprecipitations. The immunoprecipitation of AURKB contained less ubiquitin in VRK1 depleted extracts (Fig. 9a, left); and consistently, the precipitation of ubiquitin contained less AURKB (Fig. 9a, right). Next, we determined the stability of AURKB protein in cycloheximide-treated cells that were transfected with empty vector or VRK1. In the control experiment without transfected VRK1 (Fig. 9b, left), AURKB has a half-life of approximately 4 h. In the VRK1 overexpression of experiment (Fig. 9b, right), VRK1 caused a significant stabilization of AURKB, with no change in level after 24 h, which is consistent with protection of AURKB against its known degradation by ubiquitylation [37].

## Discussion

The dynamic reorganization of chromatin occurring in the progression of the cell cycle depends on the local pattern of histone modifications. These histone modifications determine the incorporation of different proteins associated with a large variety of biological processes [39–41], ranging from transcription to replication, DNA repair, chromosome condensation, and relaxation, among others. The changes in chromatin reorganization might be either global or local depending on their specific functions. The inclusion in the complexes of kinases associated with chromatin permits the integration and coordination of multiple sequential changes in chromatin structure and function. VRK1 role in the regulation of chromatin organization and associated functions can vary depending on local protein interactions and the stage of the cell cycle. VRK1 as a chromatin kinase plays a role in the regulation of transcription factors [42–44], and in chromatin reorganization in DNA-damage responses [26, 33, 45–47]. Therefore, the formation of a stable protein complex, formed by VRK1 and AURKB, is a likely player in different temporal and functional aspects of chromatin remodelling during mitosis. Their functions are associated with the effect on their phosphorylation targets, as well as on the recruitment of proteins involved in different mitotic functions. However, the inhibition of protein kinase activity in the context of chromatin remodelling is not well understood. The cross inhibition of VRK1 and AURKB kinase activity, when forming a complex, can be relevant at specific chromatin locations. Therefore, the location of the kinases is critical to determine their activity, which is modulated by both protein interactions and covalent modifications.

VRK1 regulates histones and its covalent modifications [26], which are essential for correct chromatin assembly. The activation of VRK1, in response to mitogenic signals [4], phosphorylates BAF [11] to facilitate nuclear envelope disassembly and also phosphorylates histone H3 in Thr3 [9], which cause the dissociation of VRK1 from chromatin and at the same time causes a global condensation of chromatin [9]. Chromosomes need to be condensed and chromatin compacted to enter mitosis, and is associated with the initial chromatin compaction linked to H3 phosphorylation by VRK1 in G2/M [9]. Histone H3 phosphorylation in Thr3 is also performed by haspin [17], but haspin participation is required for chromosome alignment in metaphase, and thus, it is temporally posterior in mitosis. Thus, it is very likely that the pattern of histone H3 phosphorylation depends on the position of the individual histone H3 molecule and the kinases present at these locations. VRK1 phosphorylates both H3–Thr3 and H2A–Thr120 [48]. Phosphorylated H3–Thr3 is recognized by survivin [25], whose gene expression is also activated by VRK1 [23]. Therefore, general H3-Thr3 phosphorylation can serve as an interaction platform for recruiting other proteins, such as AURKB that recruits the phosphatase PP1, and resulting in a local dephosphorylation of H3-Thr3 [49, 50]. The previous phosphorylation of H3T3 is necessary for the recruitment of AURKB to centromeres [25] and the formation of the chromosomal passenger complex [25]. Two histone marks are required for the establishment of the inner centromere and chromosome bi-orientation [51]. The loss of these phosphorylations can account for the delocalization of ACA and AURKB that was observed when VRK1 was depleted, and is consistent with the role of VRK1 as an early participant in mitosis, which is also needed for initial chromatin compaction in G2/M [9]. After the formation of the complex formed by AURKB–H3T3ph in condensed chromatin, this local dephosphorylation of H3 can be done by either MKP2 (DUSP4) recruited by VRK1 [52], and PP1 recruited by AURKB [49, 50]. Then, the AURKB–H3T3ph complex after its dephosphorylation recruits haspin, a kinase that rephosphorylates H3–Thr3 [22] at the specific locations where AURKB is already present in centromeres, and which is required for metaphase alignment [14]. This also facilitates a phosphorylation of H3 in Ser10 by AURKB, in a different and local context, and allows the progression of chromosome dynamics in mitosis [19, 22, 53]. Thus, the effect of VRK1 on H3–Ser10 phosphorylation is indirect, since it is not a direct target [26], and a consequence of the effect of VRK1 on recruitment of other kinases, such as AURKB. AURKB is an important middle step in mitosis by preventing both late replication and early exit from mitosis [54]. Alternatively, inhibition of AURKB activity can also be a mechanism to facilitate its accessibility to ubiquitin ligases that participate in its downregulation at the end of mitosis, such as the APC/C complex [55, 56] or Cullin3 [57].

**Fig. 10:**
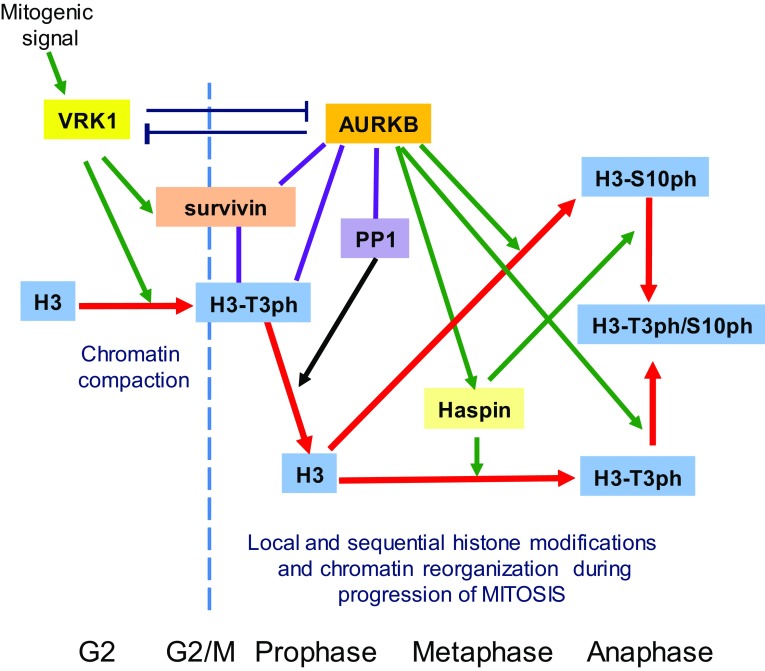
Model of the sequential organization of VRK1 and AURKB in mitotic progression and phosphorylation of Histone H3. The red arrow indicates the fate of histone H3 and its phosphorylation as mitosis progresses. The lines indicate an interaction. Green arrows indicate an effect, either phosphorylation or gene expression in the case of survivin. *PP1* phosphatase 1

The temporal coordination of VRK1 and AURKB roles in chromatin reorganization can be envisaged as a sequential process involving sequential histone H3 phosphorylations (Fig. 10). Initially, VRK1 phosphorylates Thr3 in chromatin to facilitate its compaction [9], and this H3–T3ph interacts with surviving and recruits AURKB to centromeres, whereby recruiting PP1/Repo-man locally dephosphorylates Thr3 [50, 58]. AURKB directly activates and phosphorylates haspin, which rephosphorylates H3 in Thr3 [14, 19]. These newly and locally rephosphorylated H3, in combination with Ser10 phosphorylation, control the transition from a general pattern of H3 phosphorylation to a localized pattern. These changes in phosphorylation patterns have functional consequences, such as the positioning of AURKB in centrosomes [22, 53], playing a role in metaphase [14, 16, 17] and facilitating the segregation of chromosomes [19], and in which haspin plays a downstream role [18]. The effect of VRK1 depletion on the centromeric localization of AURKB might be indirect and a consequence of its effect on chromatin organization. Moreover, AURKB precedes haspin, since the activating phosphorylation of haspin by AURKB is necessary for the phosphorylation of H3T3 by haspin in mitosis [53]. An important issue that has not yet been properly addressed is the role played by the different H3 variants [59–61] in the sequential processes associated with the progression of mitosis. Most of the work on H3 phosphorylation has been studied independently of the H3 variant implicated, but the variant might be different for each kinase, something that will require further studies. This sequential control of two histone marks is consistent with their requirement for the establishment of inner centromere and chromosome bi-orientation [51].

The protective effect of VRK1 on the stability of AURKB is in agreement with a similar effect of VRK1 on p53 [31], coilin [28] or NBS1 [36]. In all of them, VRK1 protects these proteins from ubiquitin-mediated degradation. Therefore, a likely role of phosphorylations mediated by VRK1 is a common and protective effect on proteins that are known to be downregulated by different ubiquitin ligase, and thus participates in the coordination of these processes. The inhibition of AURKB activity might also contribute to facilitate its ubiquitination and degradation at the end of mitosis [57, 62].

The complex between VRK1–AURKB can have additional roles in cell cycle progression by the regulation of p53 and affecting mitotic checkpoints [63, 64]. Each kinase has a different role. VRK1 activates p53 by phosphorylation of the N-terminus [31], while AURB phosphorylates the C-terminus of p53 to facilitate its degradation by ubiquitination [34]. The peak in the formation of the VRK1–AURB complex at the end of mitosis, and the cross inhibition of their activities can be a downregulatory signal for the completion of cell division and will require further studies.

We conclude that the three kinases phosphorylating histone H3 in mitosis have a different temporal and spatial order, and contribute to the coordination of the sequential and dynamic changes in chromatin from its initial condensation, to the redistribution of chromosomes into daughter cells.

## Materials and methods

### Cell lines

The validated cell lines (ATCC) used in this work were grown in the respective culture medium recommended by the supplier. HEK293T and HeLa cells were used for transfection and interaction analysis; U2OS cells were used for cell cycle synchronization and confocal microscopy analysis. Cells were grown in DMEM medium with 10% fetal bovine serum, 2 mM l-glutamine, and antibiotics penicillin (50 units/mL) and streptomycin (50 μg/mL) [28, 47].

### Cell cycle analysis

Initially, cells were seeded in 10 mm *Style* cell culture dishes and incubated with the appropriated treatment. Then, cells were fixed in 70% ethanol in 1× phosphate buffered saline (PBS) for 30 min. After the removal of the fixation agent, through centrifugation, the cells were stained with a solution of 0.5% propidium iodide and 0.5% RNAase in 1× PBS for 1 h, in the absence of light. Finally, the cells were acquired (20,000 events) in a FACSort Cytometer (Becton-Dickinson; Franklin Lakes, NJ, USA), and the analysis of the results was performed using both Paint-a-Gate (Becton-Dickinson; Franklin Lakes, NJ, USA) and ModFit software (Verity Software House; Topsham, ME, USA).

### Plasmids

Expression of VRK1 in mammalian cells: pCEFL–HA–VRK1 (HA–VRK1). Kinase-dead VRK1: pCEFL–HA–VRK1–K179E (HA–VRK1–KD) [12, 32]. Expression of VRK1 in *E. coli*: pGEX–GST–VRK1, pGEX–GST–VRK1 (K179E), pGEX–p53 (1–85) [12, 33, 35]. Expression of AURKB in mammalian cells pDEST3.1_nV5–AurkB (AURKB), pDEST3.1–V5–AurkB (K106R) (AURKB–KD, kinase-dead) Expression of AURKB in *E. coli*: pGEX–GST–AurkB, pGEX–GST–AurkB (K106R) (kinase-dead) all provided by Malumbres [65]. Murine VRK1 plasmids: pCMV6–myc–mVRK1 or its kinase-dead mutant pCMV6–myc–mVRK1 (K179E) [28]. Ubiquitin was expressed with plasmid pcDNA3–6xHis–Ubiquitin (wt) (J. Lozano, University of Malaga, Spain) [66].

### Cell transfections

The overexpression of proteins was done by cell transfection with DNA plasmids as previously reported with JetPEI [28, 36, 47].

### RNA interference

The suppression of VRK1 expression was done using specific siRNA from *Dharmacon RNA Technologies* (Dharmacon, Inc.; Lafayette, CO, USA) or *OriGene Technologies* (Rockville, MD, USA), respectively. Specific silencing of VRK1 was performed using two different siRNA: siVRK1-02 (siV1-02) and siVRK1-03 (siV1-03) from Dharmacon (DHARMACON RNA Technologies). The sequence target of the two VRK1 siRNA oligos was siVRK1-02: CAAGGAACCTGGTGTTGAA and siVRK1-03: GGAAUGGAAAGUAGGAUUA. As negative control, indicated as siCt in experiments, the “ON-TARGETplus siCONTROL Non-targeting siRNA” from DHARMACON was used. The efficiency of RNAi transfection was determined with “siGLO RISC-free siRNA” (DHARMACON). Briefly, cells were transfected with the indicated siRNA at a concentration of 20 nM using Lipofectamine 2000 Reagent (Invitrogen) according to the manufacturer’s instructions. After transfection, cells were processed at the times indicated in specific experiment that were performed as previously reported [28, 45, 47, 67].

### VRK1 rescue experiments

Rescue of defective of histone phosphorylation and localization of survivin was determined using murine VRK1 (mVRK1) constructs resistant to siRNA. U2OS cells were transfected with siVRK1-02 to knock down endogenous human VRK1, and later were retransfected with murine VRK1 (plasmid pCMV6–myc–mVRK1 or its kinase-dead mutant pCMV6–myc–mVRK1 (K179E) [28]. Thirty-six hours after retransfection, cells were immunostained with anti-myc polyclonal antibodies to identify cells expressing exogenous mVRK1. Efficiency of endogenous human VRK1 silencing was determined by Western blot.

### RNA extraction, purification, and qRT-PCT

The cells were seeded in 10 mm *Style* cell culture dishes. Cells were scraped and centrifuged. The cell pellet was resuspended in 1 mL of TRIzol (Invitrogen-Life Technologies; Carlsbad, CA, USA), for 5 min incubation. Next, the mixture was careful homogenized with chloroform (Merck; Whitehouse Station, NJ, USA), and after 2 min of incubation at RT, the tubes were centrifuged at 4 °C at 13,500×*g* during 15 min. The upper RNA phase was transferred to a new Eppendorf and 2-propanol (Merck) was added to the mixture. Afterwards, the tubes were incubated for 10 min on ice and centrifuged at 4 °C, at 13,500×*g,* during 10 min. Finally, the supernatant was eliminated, 75% ethanol (Sigma-Aldrich) was added, and after a 5 min centrifugation at 4 °C at 13,500×*g*, the pellet was resuspended in RNase-free water (Qiagen; Venlo, The Netherlands).

Purification of RNA was performed with the *RNeasy*^*®*^ *Mini Kit* (Qiagen), following the manufacturer’s instructions. Briefly, the extracted RNA was homogenized in lysis buffer complemented with β-mercaptoethanol (Sigma-Aldrich) and 100% ethanol (Sigma-Aldrich). Then, the complete volume was transferred to a RNA-purification column and centrifuged at 9350×*g* for 15 s at RT. Next, the RNA was washed with distinct *washing membrane*-*bound RNA* buffers (9350×*g* for 15 s at RT), through the RNA-purification column, and finally, the RNA was eluted in RNase-free water (Qiagen). The RNA was quantified with the spectrophotometer NANOdrop (NanoDrop Technologies; Wilmington, DE, USA), and the 260/280 nM and 260/230 nM ratios were analyzed to evaluate the quality of the RNA. The qRT-PCR was prepared using 100 ng of the purified RNA (diluted at 50 μg/μL). The primers used for qRT-PCR were: survivin (forward: 5′-AGGACCACCGCATCTCTACAT-3′, and reverse: 5′-AAGTCTGGCTCGTTCTCAGTG-3′) and GAPDH (forward: 5′-GGTCTTACTCCTTGGAGGCCATGTG-3′ and reverse: 5′-ACCTAACTACATGGTTTACATGTT-3′) as an internal control [68]. The reactions was performed with the kit *iScript™ One*-*Step RT*-*PCR Kit With SYBR*^*®*^ *Green* (Bio-Rad) in a total volume of 25 μL in a *iCycler* thermocycler (Bio-Rad). Data were analyzed using the Bio-Rad iQ5 software (Bio-Rad) [47].

### Antibodies

All the primary antibodies used in this work are listed in Table 1.

**Table 1:** Antibodies used in this work

Antibody	Type	Dilution (WB/IF)	Clone and/or reference	Supplier
ACA	Human	–; 1:200	15-235-0001	Antibodies Inc., San Diego, CA, USA
AURKB	Rabbit monoclonal	1:1000; 1:100	ab45145	Abcam
AURKB	Rabbit polyclonal	1:100	ab2254	Abcam
Cyclin D1	Rabbit polyclonal	1:1000	M27/sc-718	Santa Cruz
Flag-Tag	Rabbit polyclonal	1:1000	F7425	Sigma-Aldrich
Survivin/BIRC5 (human)	Mouse monoclonal	–; 1:100	3F5H5/37-2000	ThermoFisher
Flag-Tag (M2)	Mouse monoclonal	1:1000	M2/F1804	Sigma-Aldrich
Flag-Tag	Mouse monoclonal	1:1000	M5/F4042	Sigma-Aldrich
GST-Tag	Mouse monoclonal	1:1000	B14/sc-138	Santa Cruz
HA-Tag	Rabbit polyclonal	1:1000	H5908	Sigma-Aldrich
HA-Tag	Mouse monoclonal	1:1000	F7/sc-7392	Santa Cruz
Histone H3	Rabbit polyclonal	1:1000	9175	Cell Signaling
Myc-Tag	Mouse monoclonal	1:1000	4A6/05-724	Millipore
Myc-Tag	Rabbit polyclonal	1:1000	06-549	Millipore
P53	Mouse monoclonal	1:500	DO1/sc-126	Santa Cruz
P53	Mouse monoclonal	1:1000	Pab1081/sc-98	Santa Cruz
P53-T18ph	Rabbit polyclonal	1:1000	ab30659	Abcam
Phospho-histone H3 (Ser10ph)	Mouse monoclonal	1:500/1:100	04-817	Millipore
Phospho-histone H3 (Thr3ph)	Mouse monoclonal	1:500/1:100	05-746R	Millipore
Phospho-histone H3 (Thr3ph)	Rabbit polyclonal	1:500/1:100	07-424	Millipore
Phospho-Rb (Ser807/811)	Rabbit polyclonal	1:1000	9308	Cell Signaling
Rb	Rabbit polyclonal	1:1000	C15/sc-50	Santa Cruz
V5-Tag	Mouse monoclonal	1:1000	ab27671	Abcam
V5-Tag	Rabbit polyclonal	1:1000	G14/sc-83849	Santa Cruz
VRK1	Mouse monoclonal	1:1000/1:200	1B5	[74]
VRK1	Mouse monoclonal	1:1000/1:200	1F6	[74]
VRK1 (N-Term)	Rabbit polyclonal	1:1000/1:200	HPA000660	Sigma-Aldrich
VRK1 (VC)	Rabbit polyclonal	1:1000	45	[74]
VRK1 (VE)	Rabbit polyclonal	1:1000	45	[74]
β-Actin	Mouse monoclonal	1:1000	AC15/A5441	Sigma-Aldrich

### Immunofluorescence and confocal microscopy

Confocal microscopy immunofluorescence (IF) was performed as previously described [28, 33, 47, 69]. The secondary antibodies were linked to the cyanine fluorophore Cy2 or Cy3 (Jackson ImmunoResearch; West Grove, PA, USA). DNA was counterstained with DAPI (Vector Labs; Burlingame, CA, USA) and coverslips were mounted in microscope slides using *MOWIOL* (Calbiochem; Billerica, MA, USA). Fluorescence images were acquired with a *Leica TCS SP5* confocal laser scanning microscope (Leica Microsystems; Wetzlar, Germany) connected to a digital video camera *Leica DC100* (Leica Microsystems). The analysis of the confocal microscopy images was performed using the *LAS AF Lite* program version: 2.6.0.7266 (Leica Microsystems) [28, 47].

### Purification of glutathione *S*-transferase fusion proteins and pull-down assays

The expression and purification of glutathione *S*-transferase (GST)-tagged proteins was performed with the resin Glutathione Sepharose 4B as previously reported [6, 27, 28, 45, 70]. The expression and purification of the protein was performed using the *E. coli* Bl21DE3 strain that was transformed with a pGEX–4T–GST plasmid containing the protein to express (pGEX4T–VRK1). Protein expression was induced with isopropyl β-d-1-thiogalactopyranoside (IPTG). Briefly, the bacteria expressing the GST-tagged proteins were preincubated overnight at 37 °C, in LB medium with ampicillin (50 μg/mL). Then, the preinoculum was diluted in fresh LB with ampicillin (1:10 dilution) and this dilution incubated at 37 °C under agitation, until it reached an optical density between 0.6–0.8 at 600 nm. The induction of protein expression was performed by adding 0.2 mM IPTG (Roche Applied Science) to the culture, and incubated at 37 °C for 2–4 h. Next, the bacteria were centrifuged at 4000×*g*, during 10 min, and the pellet resuspended in lysis buffer (1% Triton X-100, 0.2 μg/mL lysozyme, 1 mM phenylmethylsulfonyl fluoride (PMSF), 5 mM dithiothreitol (DTT), 10 μg/mL aprotinin, and 10 μg/mL leupeptin in 1× PBS). The suspension was submitted to sonication using the sonicator *Misonic XL2010* (Misonix Inc.; Farmingdale, NY, USA), performing 3–5 short “10 s” bursts, alternated with “10 s” on ice. Next, the sonicated cell lysate was incubated at 4 °C, for 30 min and centrifuged, at 10,000×*g*, for 30 min. The soluble fraction was incubated overnight with *Glutathione Sepharose 4B beads* (GE Healthcare; Buckinghamshire, UK). The resin was washed several times with 1× PBS, with proteases inhibitors, at 400×*g*, during 3 min (4 °C), and the GST-tagged protein eluted from the resin with a solution of 10 mM reduced-glutathione in 50 mM Tris–HCl (pH 8.0), at 4 °C, during 4–12 h in a rotator. The eluted protein was separated from the resin by centrifugation, at 400×*g* for 3 min. The purification of GST-tagged protein was confirmed by sodium dodecyl sulfate polyacrylamide gel electrophoresis (SDS-PAGE) followed by Coomassie blue staining or Western blot (WB). The protein levels were quantified by colorimetric assay (*Bio*-*Rad protein assay,* Bio-Rad), using bovine serum albumin (BSA) to generate the standard curve.

### Cell lysates

The protein extracts were obtained using two different lysis buffers: composition of Suave lysis buffer (50 mM Tris–HCl (pH 8.0), 1 mM EDTA, 150 mM NaCl, and 1% triton X-100) and RIPA lysis buffer (150 mM NaCl, 1.5 mM MgCl_2_, 10 mM NaF, 4 mM EDTA, 50 mM Hepes, 1% triton X-100, 0.1% SDS, and 10% glycerol) [26, 28, 71]. At the time of the lysis, the buffers were complemented with phosphatases inhibitors (1 mM NaF and 1 mM sodium orthovanadate) and proteases inhibitors (1 mM PMSF, 10 μg/mL aprotinin, and 10 μg/mL leupeptin).

### Acid extraction of histones

Histones were acid extracted from HeLa cells as reported [26, 72]. Briefly, cells (3 × 10^6^ cells/mL) were collected and washed with 1× PBS. Cells were lysed in a hypotonic lysis buffer (10 mM Tris–HCl, pH 8.0; 1 mM KCl (potassium chloride); 1.5 mM MgCl_2_; 1 mM DTT, supplemented with proteases and phosphatases inhibitors) for 30 min, at 4 °C, on rotation. Intact nuclei were recovered by centrifugation, at 4 °C, (10 min, 10,000×*g*), and resuspended in 0.4 N H_2_SO_4_ (sulfuric acid). Samples were centrifuged (10 min, 16,000×*g*), at 4 °C, and the supernatant containing the histone fraction was collected. Histones were precipitated with trichloroacetic acid and recovered by centrifugation, at 4 °C (10 min, 16,000×*g*), and the pellet-containing histones were washed with ice-cold acetone, at 4 °C (5 min, 16,000×*g*). The histones posttranslational modifications were analyzed by electrophoresis in 12.5% SDS-PAGE for WB.

### SDS-PAGE electrophoresis and western blot

The separation of proteins was performed, accordingly to their size, in denaturing conditions, through SDS-PAGE vertical electrophoresis. Proteins were transferred to PVDF *Immobilon*-*P* or *Immobilon*-*FL* membranes (Millipore) as previously reported [28, 36, 47, 67]. PDVF membranes were incubated with the specific primary antibody (Table 1). The secondary antibodies *Goat Anti*-*Mouse IgG, DyLight 680* (red colored) and *Goat Anti*-*Rabbit IgG, DyLight 800* (green colored) (Thermo Scientific) were incubated at 1:10,000 dilutions in TBS-T, during 1 h (in the dark). Next, membranes were washed with TBS-T (3×, 10 min washes) and scanned in the *LI*-*COR Odyssey Infrared Imaging System* (LI-COR Biosciences; Lincoln, NE, USA) that detects the fluorescence associated with the secondary antibody. The secondary antibodies were either Anti-Mouse IgG-Horseradish Peroxidase-Linked Species-Specific Whole Antibody (Amersham Biosciences; Amersham, UK) or Anti-Rabbit IgG-Peroxidase Conjugate (Sigma-Aldrich). In these cases, after a 1 h incubation with the secondary antibody (1:10,000), the luminescence was detected with the *ECL Western Blotting Detection Reagent*.

### Immunoprecipitation

Immunoprecipitation (IP) was performed using between 0.5 to 2.0 mg of total protein extracts, in a 1 mL total volume. Protein extracts were incubated with the equilibrated resin *Gammabind plus Sepharose* (GE Healthcare). The method has been reported previously [26, 28, 36, 47].

### In vitro kinase assay

The Ser-Thr kinase activity of either VRK1 or AURKB was performed under the same experimental conditions through in vitro kinase assays using radiolabeled ATP [γ-^32^P] [6, 27, 47]. This technique was performed using either GST-fusion kinase proteins expressed in *E. coli* or immunoprecipitated (endogenous or transfected) protein kinase from cell extracts. The kinase assay buffer contained 20 mM Tris–HCl (pH 7.5), 5 mM MgCl_2_, 0.5 mM DTT and 150 mM KCl), 5 μM cold ATP, 5 μCi (0.1 μM) [γ-^32^P] ATP, and a specific substrate, such as recombinant proteins (e.g., GST–p53, GST–VRK1 K179E, or GST–AURKB K106R) or commercial human histone H3 (Upstate-Millipore; Billerica, MA, USA). The conditions of the kinase assay were as previously reported [6, 28, 35]. Alternatively, under the same experimental conditions, the specific phosphorylation of histone H3 in Thr3 or Ser10 was detected using specific antibodies (Table 1).

### Database of protein interactions

The protein interactions from this publication have been submitted to the IMEx (http://www.imexconsortium.org) consortium through IntAct [73] and assigned the identifier IM-25676.

## Electronic supplementary material

Below is the link to the electronic supplementary material.

Supplementary material 1 (PDF 4478 kb)

## Abbreviations

VRK1: Vaccinia-related kinase 1
AURKB: Aurora kinase B
ACA: Anti-centromere antibody
H3: Histone 3
CPC: Chromosome passenger complex
