# Supplementary material for: VRK1 and AURKB form a complex that cross inhibit their kinase activity and the phosphorylation of histone H3 in the progression of mitosis
Source: Cell Mol Life Sci. 2018 Jan 16;75(14):2591–611. doi: 10.1007/s00018-018-2746-7 (PMC6003988; doi:10.1007/s00018-018-2746-7)
Supplement: Supplementary file 1 — Supplementary material 1 (PDF 4478 kb) [file 18_2018_2746_MOESM1_ESM.pdf]

Supplementary Information

VRK1 and Aurora kinase B form a complex that cross inhibit their kinase activity and the phosphorylation of histone H3 in the progression of mitosis

David S. Moura[1,2], Ignacio Campillo-Marcos[1,2], Marta Vázquez-Cedeira[1,2], and Pedro A. Lazo[1,2]

[1] Experimental Therapeutics and Translational Oncology Program, Instituto de Biología Molecular y Celular del Cáncer, CSIC-Universidad de Salamanca, Campus Miguel de Unamuno, 37007 Salamanca, Spain

[2]Instituto de Investigación Biomédica de Salamanca-IBSAL, Hospital Universitario de Salamanca, 37007 Salamanca, Spain

0% FBS

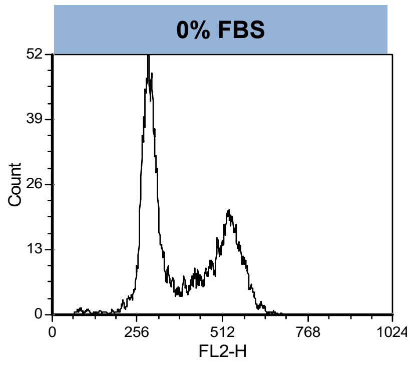

Cell Cycle Phase	%
G0/G1	51,07
G1/S	10,68
G2/M	33,68

10% FBS

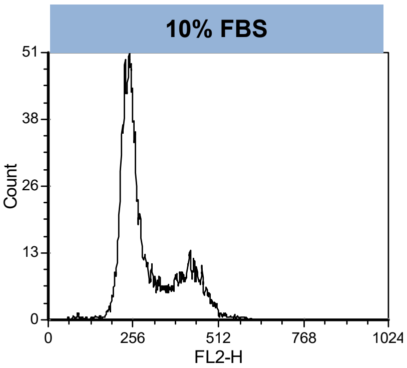

Cell Cycle Phase	%
G0/G1	65,65
G1/S	9,46
G2/M	20,43

Thymidine

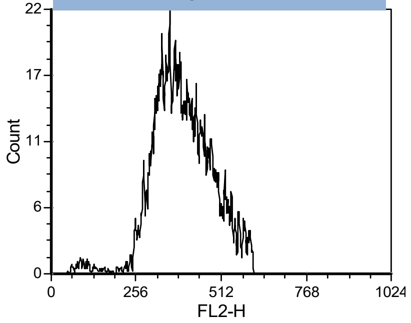

Cell Cycle Phase	%
G0/G1	45,73
G1/S	36,82
G2/M	11,55

Thymidine-Nocodazole

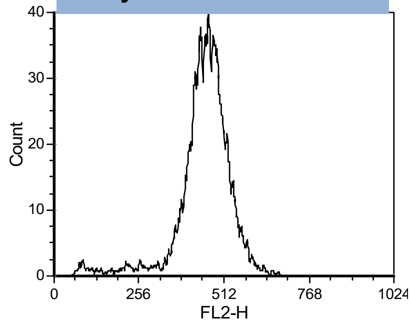

Cell Cycle Phase	%
G0/G1	2,80
G1/S	0,72
G2/M	93,48

30min release from Nocodazole

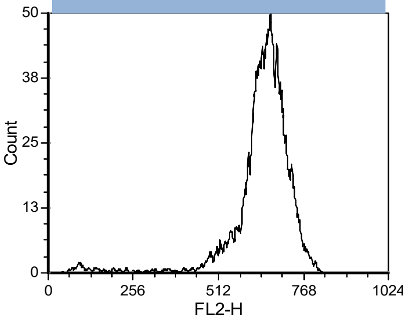

Cell Cycle Phase	%
G0/G1	1,11
G1/S	0,30
G2/M	95,75

60min release from Nocodazole

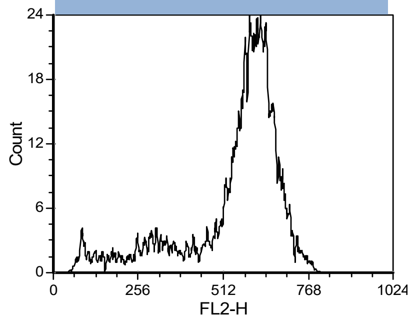

Cell Cycle Phase	%
G0/G1	10,70
G1/S	1,27
G2/M	80,50

90min release from Nocodazole

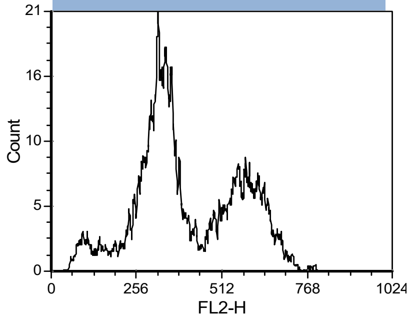

Cell Cycle Phase	%
G0/G1	50,86
G1/S	1,84
G2/M	36,18

180min release from Nocodazole

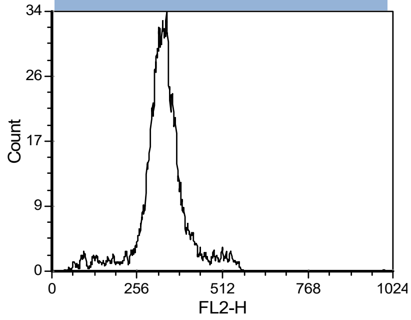

Cell Cycle Phase	%
G0/G1	87,47
G1/S	1,61
G2/M	4,81

360min release from Nocodazole

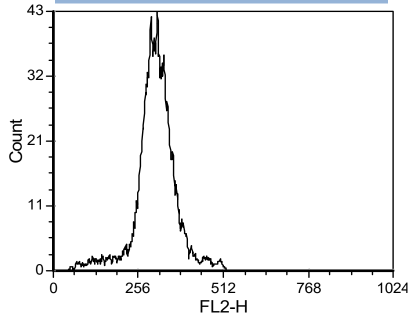

Cell Cycle Phase	%
G0/G1	92,54
G1/S	0,95
G2/M	1,42

Supplementary Figure S1. U2OS Cell Cycle by FACS. 24 hours after plate the cells, U2OS cells were treated with serum-free medium for 72 hours, to arrest the cells at G0/G1, or with double-thymidine block to arrest cell cycle at S-phase, or with double-thymidine block , followed by nocodazole treatment to arrest cells at G2/early mitosis, or subsequent release from nocodazole during 720minutes. The cells were fixed in 70% Ethanol, marked with Propidium iodide and cell cycle analyze by FACS

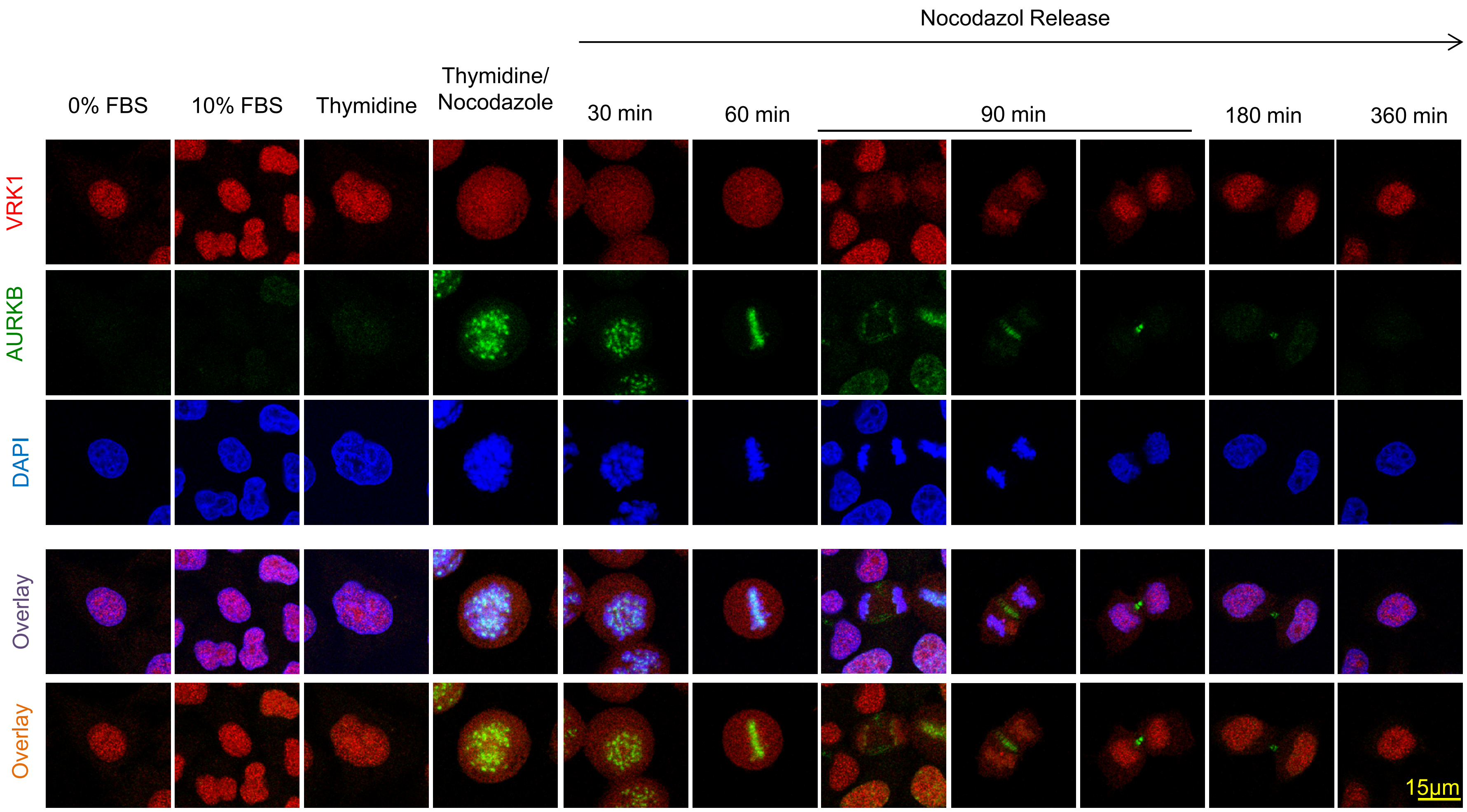

Supplementary Figure S2. The endogenous VRK1 and endogenous Aurora Kinase B, in synchronized cells, seems to have different localizations during cell cycle. 24 hours after plate the cells, Hela cells were treated with serum-free medium for 48 hours, to arrest the cells at G0/G1, or with thymidine for 24 hours to arrest cell cycle at S-phase, or with thymidine for 24 hours, followed by 12 hours of nocodazol to arrest cells at G2/early mitosis, or after thymidine and nocodazol treatment, released from arrest during 360 minutes to obtain the cells in different mitosis phases. In immunofluorescence, Aurora Kinase B was detected with rabbit monoclonal anti-Aurora-B (N-term) antibody (Epitomics,, (1:100). Human VRK1 was detected using mouse monoclonal anti-VRK1 antibody (1B5) (1:200). DAPI (1:1000).

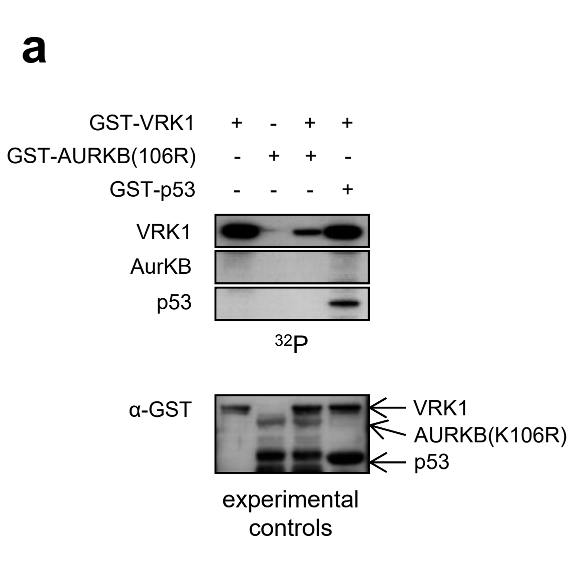

Supplementary Figure S3. a. Kinase assay with recombinant proteins GST-VRK1 and inactive GST-AURKB K106R. Proteins were incubated for 30 minutes at 30ºC in presence of 5 µM ATP and 5 µCi ^{32}P[ATP]. The phosphorylated proteins were analyzed in 10% SDS-PAGE. GST recombinant proteins were detected with a mouse monoclonal anti-GST antibody (1:1000) (Santa Cruz Technology, Inc.) and the incorporated radioactivity was detected. **b.** Kinase assay with inactive recombinant proteins GST-VRK1 K179E and endogenous AURKB. Proteins were incubated for 30 minutes at 30ºC in presence of 5 µM ATP and 5 µCi ^{32}P[ATP]. The phosphorylated proteins were analyzed in 10% SDS-PAGE. AURKB was detected with a rabbit monoclonal anti-AURKB (1:1000) (Epitomics), histone H3 was detected using a rabbit policlonal anti-histone H3 antibody (1:1000) (Cell Signaling), GST recombinant proteins were detected with a mouse monoclonal anti-GST antibody (1:1000) (Santa Cruz Technology, Inc.) and the incorporated radioactivity was detected.

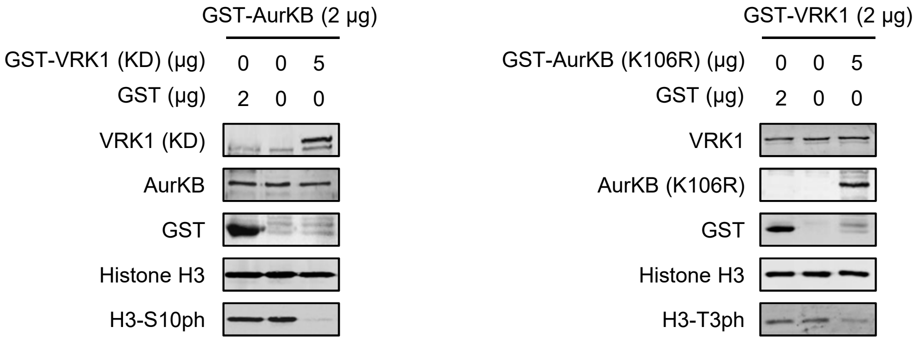

Supplementary Figure S4. GST does not affect the phosphorylation of histone H3 by either VRK1 or AURKB. A (left). VRK1 directly phosphorylates Thr3 in histone H3. B (right). AURK directly phosphorylates Ser10 in histone H3. In vitro kinase assay with cold ATP. GST-VRK1 (pGEX-GST-VRK1) or GST-AURKB (pGEX-GST-AURKB) was incubated with human histone 3 at 30ºC for 30 minutes. H3-T3p was detected using a rabbit polyclonal anti-phospho-H3T3 antibody. H3-S10p was detected using a rabbit polyclonal anti-phospho-H3S10. Total histone 3 was detected using a rabbit polyclonal anti-histone 3 antibody, and GST fusion proteins were detected using a mouse monoclonal anti-GST antibody.

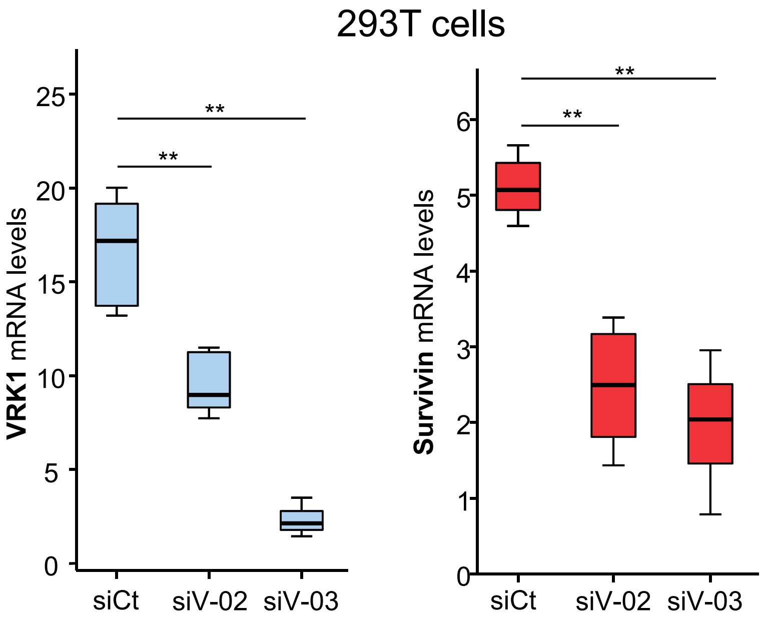

Supplementary Figure S5. VRK1 is required for expression of endogenous *BIRC5* (survivin) gene expression. VRK1 depletion cause a loss of expression of *BIRC5* (survivin) in HEK293T cells. The level of RNA was determined by qRT-PCR. Each experiment was independently performed three times. In each experiment, values were also determined in triplicates. **P<0.01. siCt: siControl; siV-02: siVRK1-02; siV-03: si-VRK1-03.

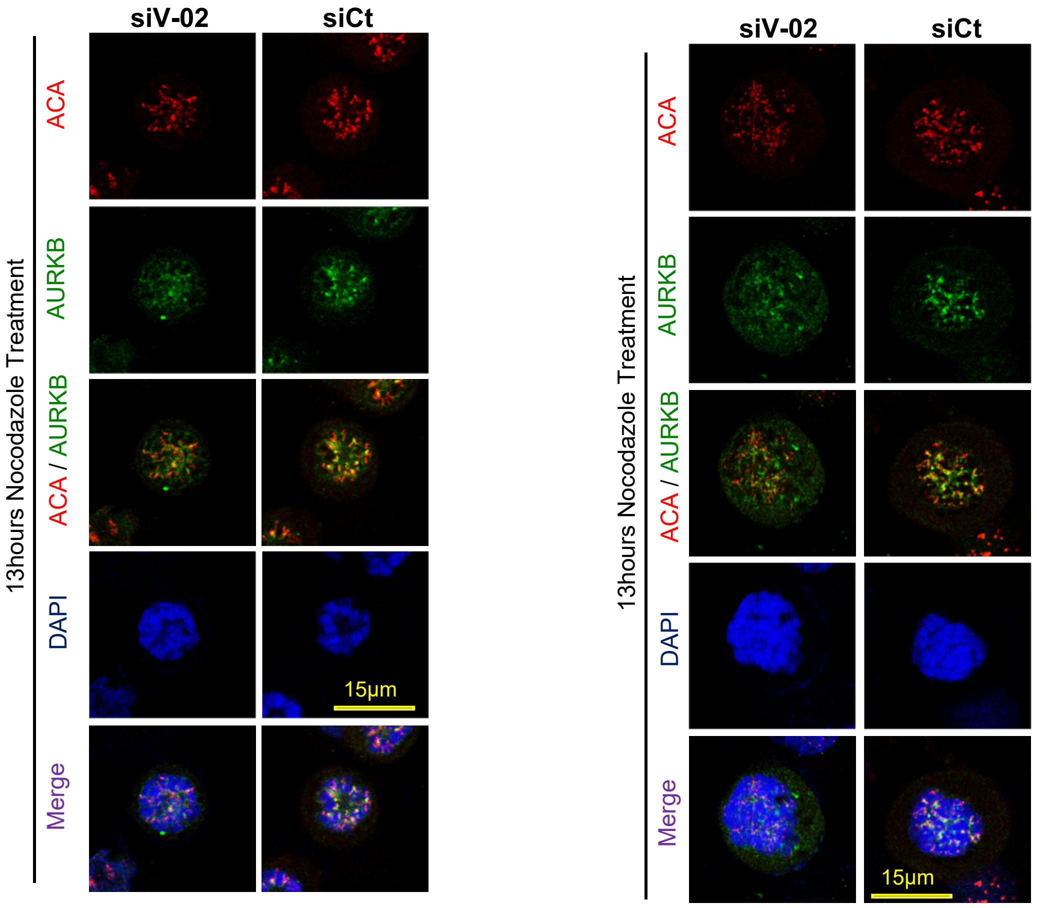

Supplementary Figure S6. Effect of VRK1 on the centromeric localization of AURKB and ACA. VRK1 in U2OS cells does not colocalize with ACA in non-synchronic cells (top panel) or in cells synchronized and arrested with nocodazole (bottom panel).

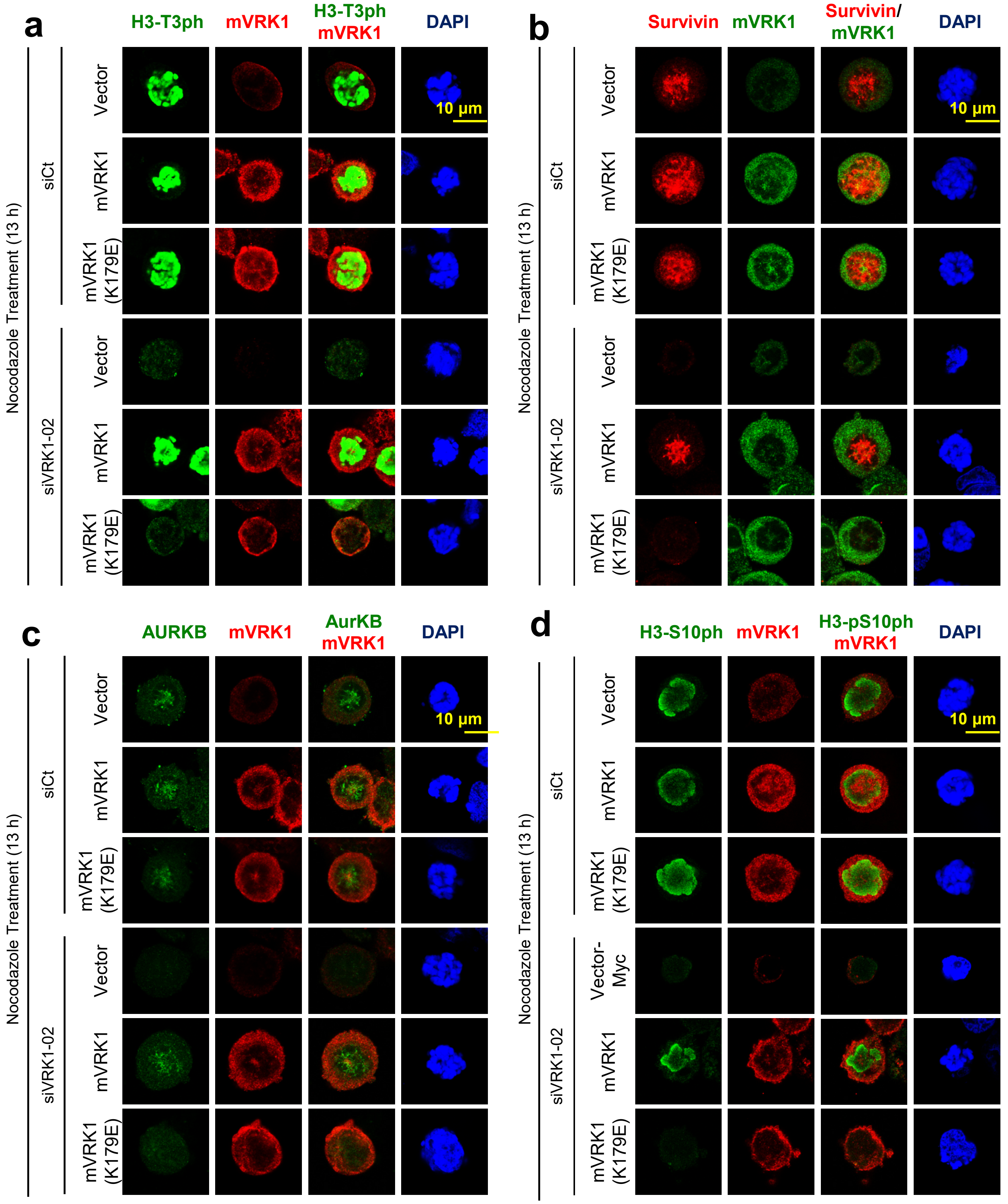

Supplementary Figure S7. Rescue of the effects regulated by VRK1. Endogenous human VRK1 was knockdown in U2OS cells that were retransfected with plasmid expressing murine VRK1 (wild-type) or its kinase-dead mutant, murine VRK1(K179E). Cell were treated with nocodazole for thirteen hours and cells fixed for determination of H3 phosphorylation in Thr3 (**a**), expression of survivin (**b**) and AURKB (**c**) and phosphorylation in Thr3 in Ser10 (**d**).
